# Scopoletin Induced Metabolomic Profile Disturbances in Zebrafish Embryos

**DOI:** 10.3390/metabo12100934

**Published:** 2022-10-01

**Authors:** Weixuan Yao, Jingpei Chen, Zhanyu Lin, Nani Wang, Anli Wang, Binjie Wang, Yuanzhao Wu, Zhongshi Xu, Jiye Wang

**Affiliations:** 1 Key Laboratory of Drug Prevention and Control Technology of Zhejiang Province, The Department of Criminal Science and Technology, Zhejiang Police College, Hangzhou 310053, China; 2 Department of Medicine, Zhejiang Academy of Traditional Chinese Medicine, Hangzhou 310012, China; 3 College of Biosystems Engineering and Food Science, Zhejiang University, Hangzhou 310058, China

**Keywords:** scopoletin, untargeted metabolomics, zebrafish, metabolic disturbances, UHPLC-Q-Obitrap-HRMS

## Abstract

Scopoletin, a typical example of a coumarin compound, exists in several Artemisia species and other plant genera. However, the systemic metabolic effects induced by scopoletin remain unclear. In the present study, we evaluated the metabolic profiles in scopoletin-exposed zebrafish embryos using UHPLC-Q-Obitrap-HRMS combined with multivariate analysis. Compared with the control group, 33 metabolites in scopoletin group were significantly upregulated, while 27 metabolites were significantly downregulated. Importantly, scopoletin exposure affected metabolites mainly involved in phosphonate and phosphinate metabolism, vitamin B6 metabolism, histidine metabolism, sphingolipid metabolism, and folate biosynthesis. These results suggested that scopoletin exposure to zebrafish embryos exhibited marked metabolic disturbance. This study provides a perspective of metabolic impacts and the underlying mechanism associated with scopoletin exposure.

## 1. Introduction

Scopoletin is a coumarin compound, which can be found in several Artemisia species and other plant genera [1], and also exist in flue-cured tobacco leaves, burley tobacco leaves, and smoke. Scopoletin plays an essential role in human health due to its antioxidant, antimicrobial, anticancer, and anti-inflammation aspects [2]. Even though a large number of studies have demonstrated the pharmacological activity of scopoletin, the toxic effects of scopoletin on human health have also attracted significant attention. Previous studies have reported that scopoletin inhibited PC3 proliferation by inducing the apoptosis of PC3 cells [3], and novel NO-releasing scopoletin derivatives induced cell death via the mitochondrial apoptosis pathway and cell cycle arrest [4]. In addition, Pan et al. [5] showed that scopoletin could inhibit vascular endothelial growth factor-induced angiogenesis through interrupting the autophosphorylation of VEGF receptor 2 and its downstream signaling pathways. In addition, scopoletin is toxic to target plants. For example, Graña et al. [6] reported that scopoletin induced strong phytotoxic effects on Arabidopsis thaliana seedlings with a mean inhibitory concentration (IC_50_) of less than 9.6 mg/L by inducing wrong microtubule assembling, mitochondrial membrane depolarization, and ultimately causing cell death. These results suggest that there is a need to explore the toxicity of scopoletin.

Zebrafish (*Danio rerio*) has been widely used as a model organism for the evaluation of toxic effects [7,8,9,10,11]. In our previous study, we found that scopoletin had developmental toxicity to zebrafish embryos and the maximum non-lethal concentration (MNLC) was 18.5 μg/mL; and that scopoletin at 18.5 μg/mL could cause varying degrees of liver degeneration, pericardial edema, muscle degeneration, yolk sac retention, and reduced the blood flow of the zebrafish embryos. However, the mechanism underlying the scopoletin-induced toxic effects and changes in metabolic profile still remain unclear. 

Recently, the rapid accumulation of metabolomic profiles has facilitated the application of comprehensive analysis of low-molecular-weight metabolites on a biological system. Metabolomics analysis can efficiently and sensitively extract and quantify a vast number of metabolites from biological samples [12]. In recent years, metabolomics has been wildly used to investigate the health effects of chemical exposure [13,14,15]. By performing untargeted metabolomics, it links metabolic pathways to biological mechanism induced by scopoletin [12]. The previous study investigated the metabolic profiling of coumarins through the combination of UPLC-MS-based metabolomics and multiple mass defect filters [14]. Moreover, Zhao et al. [16] found the metabolic map of osthole, a coumarin compound from plants, and its effect on the levels of endogenous metabolites using UPLC-ESI-QTOFMS-based metabolomics. However, according to the literature, comprehensive profiles of scopoletin-induced metabolic pathways yet remain to be fully elucidated. 

Therefore, we hypothesized that scopoletin may induce metabolic disturbances to zebrafish embryos. We used zebrafish as a model to observe scopoletin-induced metabolic changes. Zebrafish embryos were exposed to different concentrations of scopoletin. We analyzed the possible biological adverse effects in metabolic pathways through the untargeted metabolomics.

## 2. Materials and Methods

### 2.1. Chemicals and Reagents

Scopoletin (purity ≥ 98%) was purchased from Shanghai Macklin Biochemical Co., Ltd. (Shanghai, China) Acetonitrile and methanol (HPLC-grade both) were purchased from Merck (Kenilworth, NJ, USA), while formic acid (≥96%) was provided by Tedia (Fairfield, OH, USA). Dimethyl sulfoxide (DMSO) was purchased from Sigma-Aldrich. Water was purified with a Milli-Q system (Millipore, Bedford, MA, USA) and was used throughout the whole experiment, including sample treatment and instrument-based analysis. Internal standard (IS) solution was prepared with 75 ng/mL of 2-Chlorophenylalanine and 15 ng/mL of L-Methionine-d3, Phenylalanine-d5, and Citric-d4 Acid in methanol from Sigma-Aldrich. Fish water was prepared with reverse-osmosis purified water containing 5 mM NaCl, 0.17 mM KCl, 0.33 mM CaCl_2_, and 0.33 mM MgSO_4_ (Millipore, Bedford, MA, USA).

### 2.2. Zebrafish Maintenance and Husbandry

Adult 6-month-old zebrafish of wide-type AB strain (*Danio rerio*) were purchased from the China Zebrafish Resource Center and cultured in a flow-through system containing fully aerated and filtered tap water at 28 ± 0.5 °C and 14 h light: 10 h dark cycle. Embryos were obtained through the natural mating of 3 males and 2 females on 4-L mesh-bottom breeding tanks in the morning under the light stimulation. Embryos were incubated under the same conditions throughout the exposure. All procedures were conducted in accordance with the guidelines of Institutional Animal Care and Use Committee (IACUC) of the Zhejiang University, Hangzhou of China. The husbandry and treatment of the zebrafish in our study were approved by the Ethics Committee of Zhejiang University (ZJU20220333).

### 2.3. Scopoletin Exposure Setup

At 48 hpf, embryos that had no observable deformity were selected and exposed to scopoletin. Our previous study found that the 120-h MNLC of scopoletin to embryos was 18.5 μg/mL (Supplementary Materials Table S1). Therefore, the exposure concentration of scopoletin was determined based on the previous study and set at 2.1 μg/mL for the low-exposure group (1/9 MNLC), 6.2 μg/mL for the medium-exposure group (1/3 MNLC), and 18.5 μg/mL for the high-exposure group (MNLC). Scopoletin was dissolved in DMSO and diluted to concentrations of 0.21 mg/mL, 0.63 mg/mL, and 1.85 mg/mL for stock solution. Each 0.5 mL of stock solution was added to 49.5 mL of fish water in 50 mL centrifuge tube and 0.5 mL of DMSO was added as a solvent control. The exposure solution was changed every day to maintain the exposure concentration of the compound during the experiment until 120 hpf. At the end of exposure, embryos exposed to the same groups were randomized and split into six biological replicates containing 30 embryos each. These samples were rinsed with fish water three times, snap-frozen in dry ice in 1.5 mL cryogenic tubes, and kept at −80 °C until sample extraction for metabolomics analysis.

### 2.4. Metabolite Extraction

The metabolites were extracted according to the previous study [17]. In brief, zebrafish embryos were homogenized in 280 μL pre-cold methanol and 20 μL of IS solution, with three 5 mm stainless beads using a TissueLyser (Qiagen, CA, USA) at 60 Hz for 10 min. The homogenates were shaken for 10 min and centrifugated (14,000 rpm, 4 °C) for 10 min. After centrifugation, 150 μL of the upper aqueous fraction were filtered the 0.22 μm organic membrane and transferred into amber chromatographic vials with a lining tube for further LC–MS analysis. An amount of 10 μL liquid from each vial was pooled as quality control (QC) solution.

### 2.5. UHPLC-Q-Obitrap-HRMS Analysis

Untargeted metabolomics analyses were performed by injecting 5 μL of the aqueous extracts in a Dionex Ultimate 3000 UPLC system coupled with a Q-Qrbitrap-MS (Q-Exactive plus MS, Thermo Scientific, Waltham, MA, USA) using the full scan data-dependent MS/MS (ddMS2) mode. Chromatographic separation was performed on an ACQUTITY UHPLC HSS T3 column (2.1 × 150 mm i.d., 1.8 μm; Waters Corp., Milford, MA, USA). UPLC conditions, including mobile phases and gradients, were optimized based on the necessary sample analysis. Briefly, the flow rate was 0.3 mL/min and the column temperature was set to 35 °C. The mobile phases were 0.1% formic acid in Milli-Q water (solvent A) and 0.1% formic acid in acetonitrile (solvent B). The optimized gradient elution was followed: 0–3 min, 2% B; 3–15 min, 2% B to 100% B; 15–17 min, 100% B; 17–17.5 min, 100% B to 2% B; and 17.5–20 min, 2% B. The MS parameters were as follows: a sheath gas flow rate of 10 arbitrary units, a spray voltage of 3.0 kV, a capillary temperature of 320 °C, an auxiliary gas heater temperature of 30 °C, and an S-lens RF level of 50. All samples (six replicates each group, 30 samples in total) were randomly injected. The instrument performance was monitored by the injection of the QC periodically every six samples.

### 2.6. Data Processing and Pathway Analysis

Data processing was performed with Compound Discoverer 3.1 (Thermo Fisher, Waltham, MA, USA). The parameters were as follows: peak area > 1,000,000; mass tolerance ≤ 5 ppm; retention time tolerance ≤ 0.2 min. The features of a coefficient of variance higher than 30% in the quality control injections were excluded from the metabolomic analyses. After the data were derived, SIMCA software (version 14.1, Umetrics, Sweden) was used for the multivariate data analyses, including principal-component analysis (PCA) and orthogonal partial least squares discriminant analysis (OPLS-DA). The differential metabolites were selected based on following thresholds: Values of variable importance in the projection (VIP) > 1; *p*-value < 0.05; and fold change > 2 or < 0.5. Metabolites were aligned according to the positive and negative ion patterns (M + H and M − H) using the Human Metabolome Database (HMDB) (http://www.hmdb.ca/ (accessed on 12 August 2022)), Chemspider (http://www.chemspider.com/Default.aspx (accessed on 12 August 2022)), and mzCloud (https://www.mzcloud.org/ (accessed on 12 August 2022)). Metabolic pathway analysis plots, global KEGG network analysis, and Debiased Sparse Partial Correlation (DSPC) analysis were produced with MetaboAnalyst 5.0 and metabolic pathways were identified based on the Danio rerio KEGG library. The Pearson Correlation and Average Linkage were utilized as the distance metrics for hierarchical clustering.

### 2.7. Statistical Analysis

The data normalization and validation for score plot analysis was accomplished using the Pareto scaling algorithm, which measured each variable using the square root of its standard deviations. The metabolic expression was determined after a logarithmic transformation. GraphPad Prism 8.0 was employed for the statistical analysis. Data are presented as the mean ± SD. A one-way analysis of variance (ANOVA) with Tukey’s multiple range tests was used to determine the statistical significance of differences in the concentration of the metabolite among groups, which was significant when the *p* values were less than 0.05 and extremely significant when the *P* values were less than 0.01.

## 3. Results

### 3.1. Metabolomic Alteration Induced by Scopoletin Exposure

**Figure 1:**
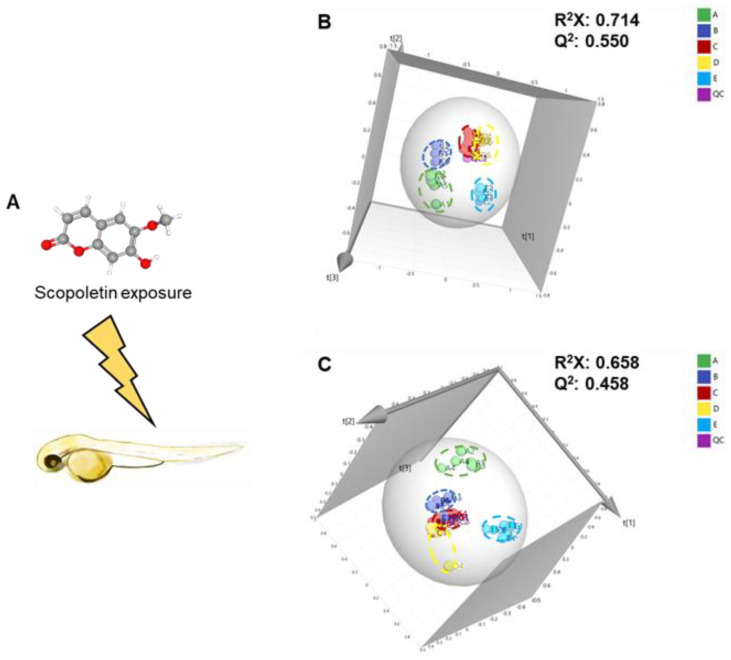
(**A**) Schematic chemical structure of scopoletin exposure. (**B**) 3D PCA score plot based on the UHPLC-MS/MS analysis in negative mode. (**C**) 3D PCA score plot based on the UHPLC-MS/MS analysis in positive mode. In the PCA model, green points represent the fish water control group, blue points represent the solvent control group, red points represent the 1/9 MNLC of the scopoletin-treated group, yellow points represent the 1/3 MNLC of scopoletin-treated group, baby-blue points represent the MNLC of scopoletin-treated group, and purple points represent the QC group.

After exposure to scopoletin, an untargeted metabolomics approach was used to fingerprint changes in the metabolomes of zebrafish embryos (Figure 1A), which may be used to learn more about the cellular chemical processes and discover new metabolite biomarkers. A total of 4705 metabolites were identified and subjected into further multivariate analyses. As shown in Figure S1 and Figure 1B,C, all the quality control samples were within the ±2 SD range in the score plot and clustered tightly in the center of the plots, indicating that the metabolomics platform had highly accuracy and reproducibility.

The PCA score plots indicated that the metabolic profiles of MNLC of scopoletin-treated group was clearly separated from the other groups, resulting in different metabolic profiles among the groups (Figure 1B,C). The quality of the established PCA models was further assessed according to R^2^X and Q^2^ parameters. The cumulative R^2^X (goodness of fit) of the supervised models established PCA model were 0.714 and 0.658 under negative and positive modes, respectively, while the Q^2^ (goodness of prediction) values were 0.550 and 0.458 given the two modes, respectively. The recorded values of R^2^X and Q^2^ for the PCA models well explain the cumulative variation of the data by the principal components and good prediction capability of the models. In addition, we have provided the PCA loading plots as Figure S2 in Supplementary Materials. In the PCA loading plots, each point represents a metabolite, and the metabolites with the labeled name represent the important compounds that caused the MNLC of scopoletin-treated group separation. Hierarchical clustering provided further evidence about the difference of metabolomes between solvent control and scopoletin-treatment groups, while the solvent control was similar to the fish water control (Supplementary Materials Figures S3 and S4). In order to further identify the segregation between the solvent control and scopoletin-treatment groups, the OPLS-DA model was used. The OPLS-DA model, which is a supervised statistical model, has a stronger classification ability than the PCA model.

**Figure 2:**
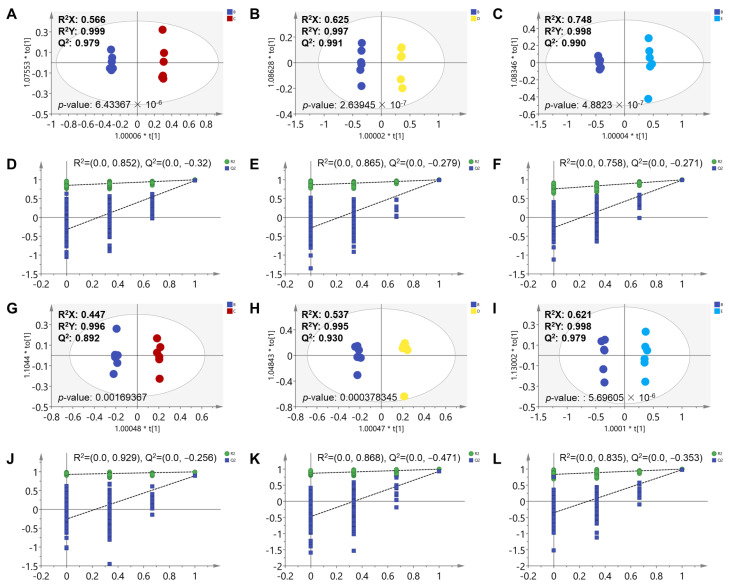
OPLS-DA (**A**–**C**,**G**–**I**) and permutation analysis (200 times) (**D**–**F**,**J**–**L**) derived from UHPLC-MS/MS analysis for the solvent control and scopoletin exposure groups. Data were acquired by negative ionization (**A**–**F**) and positive ionization (**G**–**L**). In the OPLS-DA model, blue points represent the solvent control group, red points represent the 1/9 MNLC of scopoletin-treated group, yellow points represent the 1/3 MNLC of scopoletin-treated group, and baby-blue points represent the MNLC of scopoletin-treated group.

Moreover, the OPLS-DA score plots of all data (solvent control, 1/3, 1/9, and MNLC, and fish water groups) were showed in Figure S5. A high value for R^2^X, R^2^Y, and Q^2^ indicates high explained variation and the predictive ability of an OPLS-DA model. The *p* values of CV-ANOVA indicate the good predictive ability of our established models. From the OPLS-DA models, the MNLC of the scopoletin-treated group was clearly separated from the other two scopoletin-treated groups (1/9 and 1/3 MNLC of scopoletin-treated groups). The 1/9 and 1/3 MNLC dose groups were closer to the fish water and solvent control groups. Then the OPLS-DA model was used in analysis between two groups. The OPLS-DA model demonstrated a significant difference in the metabolic profiles of the solvent control and three scopoletin-treatment groups in both negative and positive modes (Figure 2A–C,G–I; respectively). The R^2^X, R^2^Y, and Q^2^ parameters in each established OPLS-DA model were showed in the figures. A high value for R^2^Y and Q^2^ (closer to 1) indicated high explained variation and predictive ability of an OPLS-DA model, respectively. Moreover, CV-ANOVA was also used to determine the significance of the model and the CV-ANOVA p value for OPLS-DA were all below 0.05, indicating the good predictive ability of our established models. In addition, the values of R^2^ and Q^2^ (Figure 2D–F,J–L; respectively) in the permutation test indicated the good repeatability and predictability of these models. Furthermore, the VIP plots were created to specify potential differential metabolites with VIP > 1 (Figures S6 and S7), and each bar represents a metabolite in these figures. The top 30 metabolites with the highest VIP are shown on the plots.

### 3.2. Analysis of Significantly Different Metabolites

**Figure 3:**
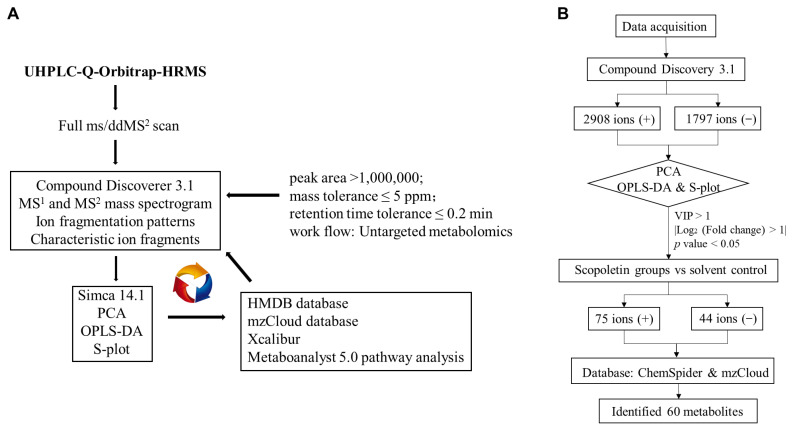
(**A**) The workflow of untargeted metabolomics approach in the current study. (**B**) Workflow of the identified differential metabolites in the untargeted analysis based on UHPLC-Q-Orbitrap-HRMS.

The metabolites with VIP > 1, *p*-value < 0.05, and the absolute of Log_2_ (fold change) > 1 were identified as potential different metabolites. As shown in the workflow (Figure 3B), 44 metabolites and 75 metabolites were selected under negative and positive modes, respectively. A total of 60 metabolites were recognized as significantly different metabolites that contributed to distinguish metabolic profiles between these groups (Supplementary Materials Table S2).

**Figure 4:**
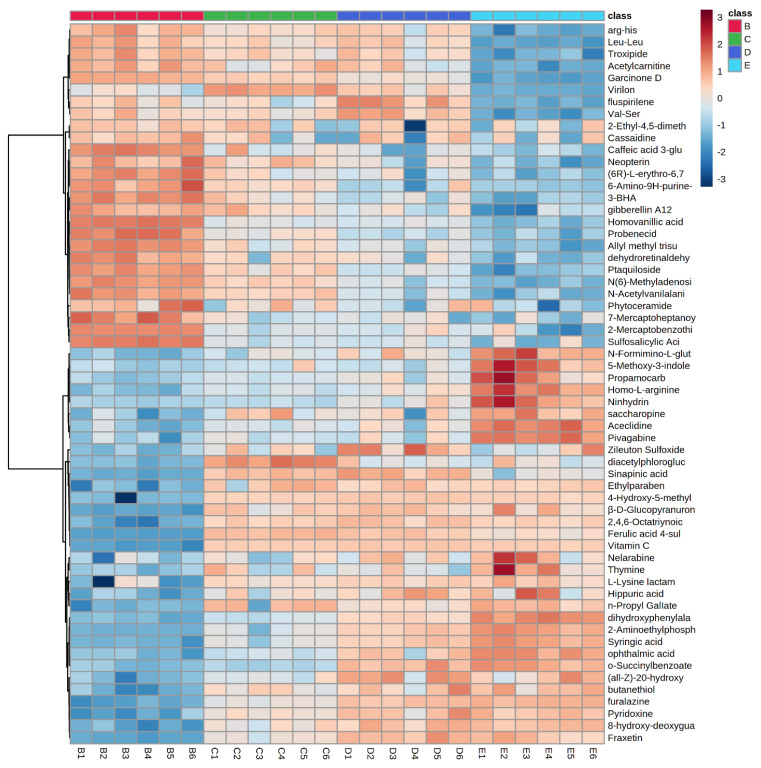
Heatmap and hierarchical clustering of differentially expressed metabolites in the solvent control and exposure groups. A 1% DMSO solvent control (group B), 1/9 MNLC of scopoletin exposure (group C), 1/3 MNLC of scopoletin exposure (group D), MNLC of scopoletin exposure (group E).

MetaboAnalyst 5.0 software was used to create the heat map of the 60 metabolites in order to intuitively spot patterns in the changes of metabolites levels between the solvent control group and scopoletin-treatment groups (Figure 4). The hue confirms a distinct contrast between the solvent control group and scopoletin-treatment groups, showing the amounts of metabolites from highest (red) to lowest (blue). 

**Figure 5:**
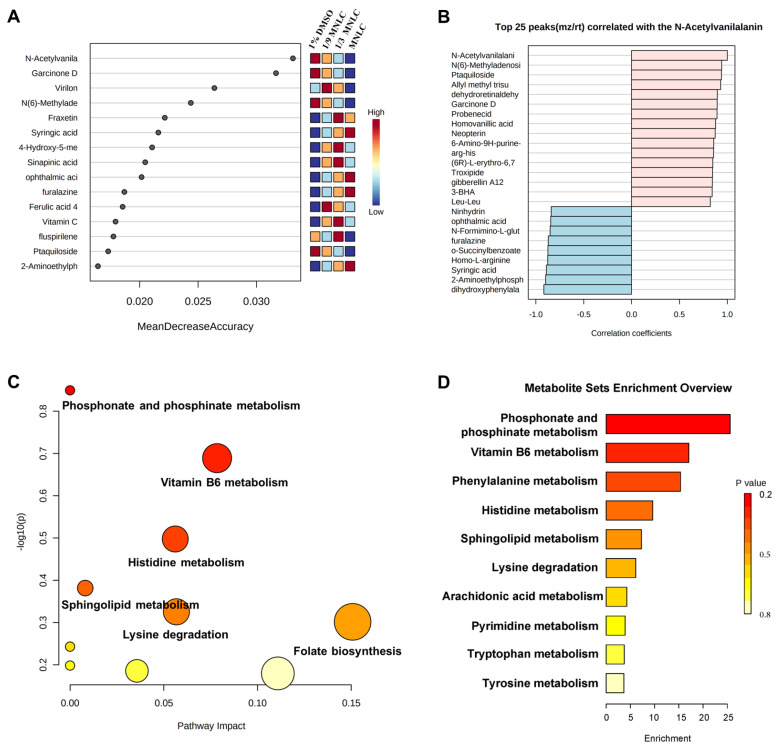
(**A**) Random forest of differential metabolites; Features ranked by their contributions to classification accuracy (Mean Decrease Accuracy). (**B**) Top 25 peaks correlated with the N-Acetylvanilalanine. (**C**,**D**) Pathway analyses with MetaboAnalyst 5.0 for the metabolic profiles of zebrafish embryos treated with scopoletin.

Compared with the control group, the intensities of 27 metabolites significantly decreased. Meanwhile, 33 metabolites were significantly elevated. Notably, we observed that these metabolites were significantly and dose-dependently changed after exposure to scopoletin (all *p* < 0.05). Among these metabolites, N-Acetylvanilalani was the most changed metabolite, and the N-Acetylvanilalani was positively changed with N (6)-Methyladenosi, Ptaquilpside, Garcinone D, Probenecid, and so on (Figure 5A,B).

### 3.3. Metabolic Pathway and Function Analysis

Metabolic profiling not only depicts changes in the level of each metabolite but also provides a thorough assessment of how harmful xenobiotics affect the metabolic process [18]. The metabolic pathway analysis of differential metabolites between the solvent control and scopoletin-treatment groups shows that the top six metabolic pathways are enriched by the KEGG library (Figure 5C,D), including phosphonate and phosphinate metabolism, vitamin B6 metabolism, histidine metabolism, sphingolipid metabolism, lysine degradation, and folate biosynthesis. With the most impacted pathways highlighted in red, scatter plots also display the matching enriched pathways from the same database according to *p*-values from the pathway enrichment analysis (*y*-axis) and pathway impacted values from the pathway topology analysis (*x*-axis) [13]. 

### 3.4. Biological Networks of Differential Metabolites

**Figure 6:**
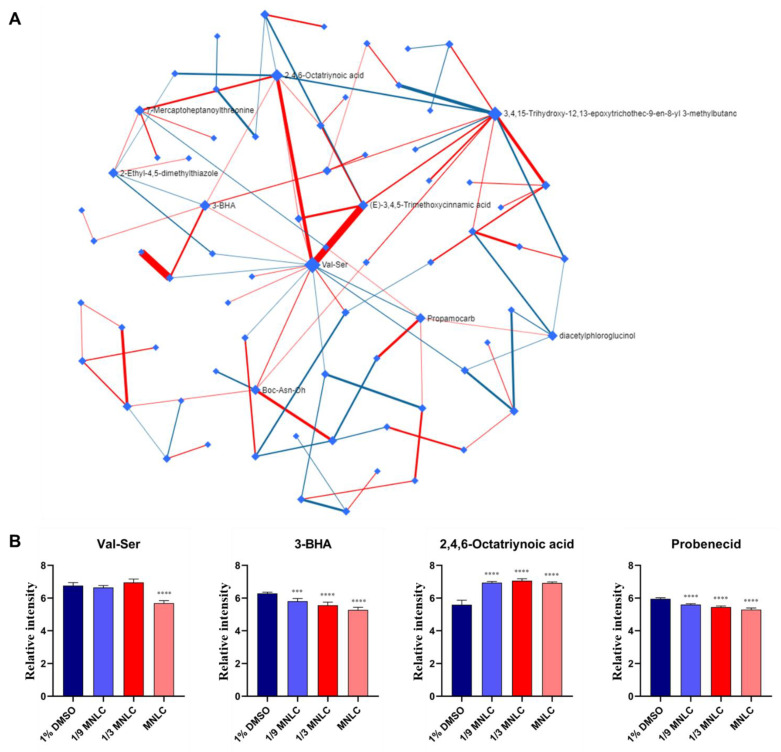
(**A**) Debiased Sparse Partial Correlation (DSPC) network of significantly differential metabolites. In the DSPC network, the nodes are input metabolites, while the edges represent the association measures. Metabolites with stronger associations cluster together and the edges between them are wider. The red lines show a positive correlation, while blue lines show a negative correlation with metabolites. (**B**) The relative concentration level of important metabolites in the DSPC network. The data were log_10_ transformed and asterisks denote statistical significance according to a multiple group comparison of ANOVA (only significative results of exposed groups vs. solvent control are illustrated); *** *p*-value < 0.001, **** *p*-value < 0.0001.

**Figure 7:**
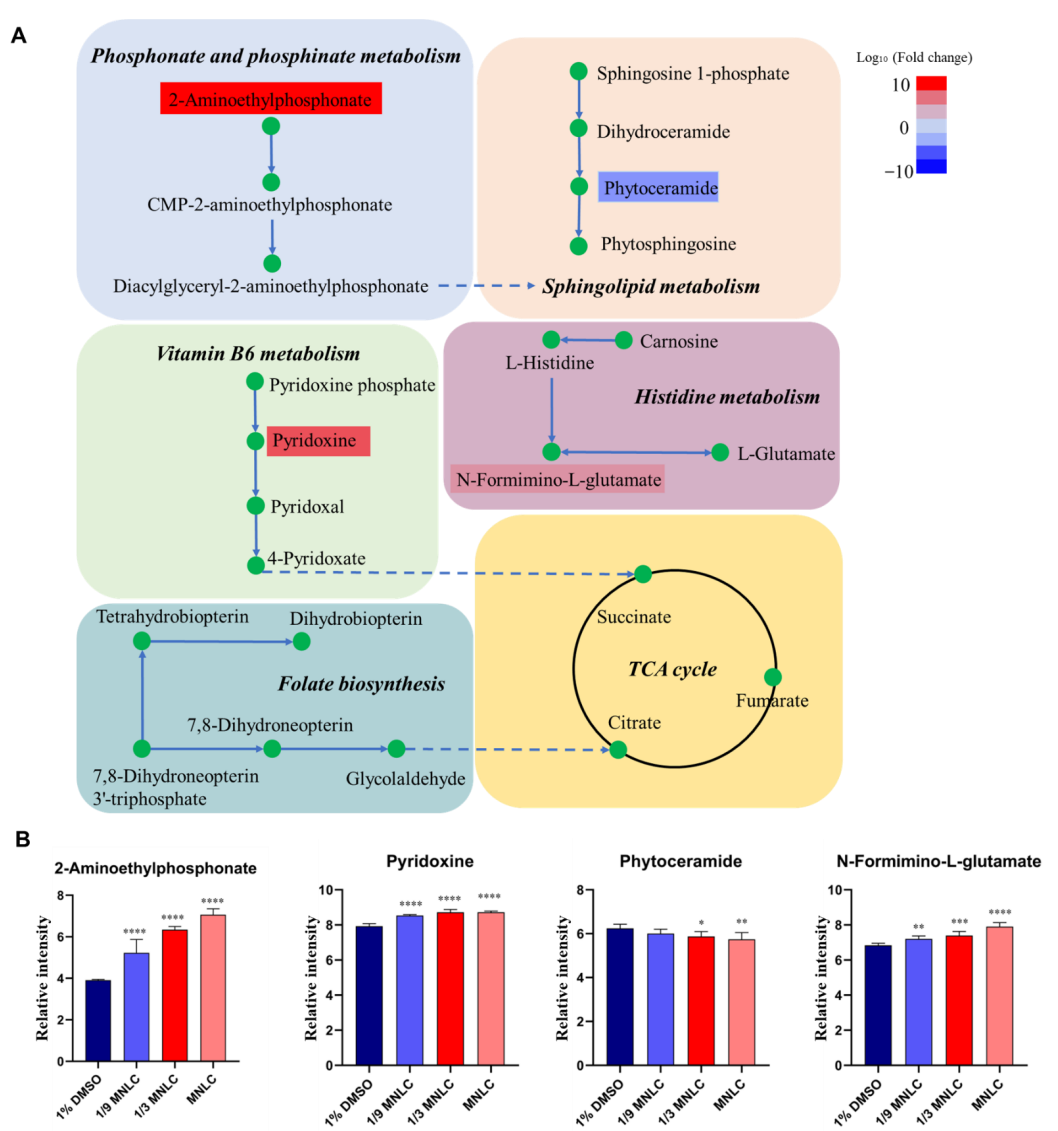
(**A**) Perturbed pathways and fluctuating metabolites in zebrafish embryos induced by scopoletin exposure. (**B**) The relative concentration level of differential metabolites in the perturbed pathways. The data were log_10_ transformed and asterisks denote statistical significance according to a multiple group comparison of ANOVA (only significative results of exposed groups vs. solvent control are illustrated); * *p*-value < 0.05, ** *p*-value < 0.01, *** *p*-value < 0.001, **** *p*-value < 0.0001.

The nodes present differential metabolites, while the lines represent the associations between these metabolites in the Debiased Sparse Partial Correlation algorithm (DSPC) network (Figure 6). For the greatest performance, the data were log or cubic root converted during the data normalization stage. The DSPC network is well suited for constructing biologically relevant networks and the identification of unknown compounds [19]. The metabolites closer to the center indicate that the greater the correlation with these selected different metabolites and the more important position in the network, such as Val-Ser. On the other hand, Val-Ser is positively associated with 3-BHA and 2,4,6-Octatriynoic acid, while negatively associated with propamocarb. In addition, the metabolic pathway network was built to determine the associations with these most perturbed pathways and differential metabolites induced by scopoletin exposure. As shown in Figure 7, 2-Aminoethylphosphonate in phosphonate and phosphinate metabolism increased depending on the concentrations of scopoletin exposure. While the level of phytoceramide, which is associated with sphingolipid metabolism, decreased in the scopoletin-exposed zebrafish embryos in a concentration-dependent manner. In the vitamin B6 metabolism, the level of pyridoxine elevated with the increasing of the scopoletin concentration. N-Formimino-L-glutamate in histidine metabolism increased in a concentration-dependent manner.

## 4. Discussion

In the present study, we evaluated the metabolic effect of scopoletin in zebrafish embryos using an untargeted metabolomics approach. The significant metabolic profile disturbance under scopoletin exposure were observed. A total of 60 metabolites were identified to be differentially expressed between the solvent control and scopoletin-exposed groups. The significantly changed metabolites between the solvent control and scopoletin-exposed groups were involved in metabolic pathways, such as phosphonate and phosphinate metabolism, vitamin B6 metabolism, histidine metabolism, sphingolipid metabolism, lysine degradation, and folate biosynthesis.

Our previous work demonstrated that scopoletin can cause developmental toxicity and affect the behavior of zebrafish embryos. The current study has demonstrated that scopoletin can cause significant metabolic profile disturbance in zebrafish embryos (Figure 1 and Figure 2). A total of 33 upregulated metabolites and 27 downregulated metabolites were identified with combining the online databases (HMDB, mzCloud, Chemspider databases) (Supplementary Materials Table S2). These significantly differential metabolites were considered as potential biomarkers of scopoletin-induced toxicity. Furthermore, these different metabolites were mainly enriched in the pathway of phosphonate and phosphinate metabolism, vitamin B6 metabolism, histidine metabolism, sphingolipid metabolism, and folate biosynthesis. These observed metabolic pathways disturbances were associated with scopoletin-induced toxic effects.

Recent studies have revealed that vitamin B6 metabolism was involved in chronic inflammation due to its antioxidant defenses in live and heart [20,21]. A dysregulation in the vitamin B6 metabolism was observed in PCB 11 exposed HepG2 cells [22] and the misonidazole neurotoxicity [23]. In accordance with these observations, our previous study found that scopoletin could induce developmental toxicity and behavioral toxicity to zebrafish. In the current study, it was postulated that the metabolic disorder of vitamin B6 metabolism associated with oxidative stress and then cause neurotoxicity to zebrafish exposed by scopoletin. 

Moreover, much research has found the association between sphingolipid metabolism and neurotoxicity [24,25,26]. Herein, sphingolipid metabolism was changed in the scopoletin treatment groups compared to that in the control group. The dysfunction of sphingolipid metabolism always occurs under oxidative stress [27]. Additionally, the exposure to scopoletin caused significant perturbations in the histidine metabolism, leading to oxidative stress responses and metabolic disturbances in energy process in the zebrafish. The above results suggested that scopoletin exposure may increase oxidative damage in the zebrafish embryos. We also found that folate biosynthesis pathway was affected in scopoletin exposure groups. Folate biosynthesis plays important role in the methylation, DNA biosynthesis and amino acids, and purine and pyrimidine synthesis. The disruption of folate metabolism is associate with cellular damage, chromosome breakage and systemic disorders, such as neurodegenerative and cardiovascular diseases [28,29]. Yin et al. [30] found that Atrazine exposure could induce significant alterations in the metabolic profiles of *C. elegans*, such as folate biosynthesis, and these changes are signs of possible oxidative stress. Taken together, these findings indicated that a metabolic disorder caused by scopoletin was likely associated with the oxidative stress of zebrafish embryos induced by scopoletin exposure. In the future, additional studies need to be carried out to confirm its mechanism.

This study indicates that scopoletin exposure was associated with significant metabolite changes in zebrafish. According to these data, we quantified a set of metabolic pathways, phosphonate and phosphinate metabolism, vitamin B6 metabolism, histidine metabolism, sphingolipid metabolism, lysine degradation, and folate biosynthesis may play critical roles in the scopoletin exposure in zebrafish embryos. Nevertheless, there were also some limitations in this study. Although metabolic profiles alteration was observed in zebrafish induced by scopoletin, the underlying mechanism of these metabolic effects change caused by scopoletin was unclear. Due to the acute exposure to scopoletin in the current study, we could not make robust conclusions about the long-term toxic effects of scopoletin. Therefore, the long-term toxic effects of scopoletin should be explored in further studies.

## 5. Conclusions

Taken together, the toxicological effects of scopoletin in zebrafish embryos were determined using a UHPLC-Q-Obitrap-HRMS-based untargeted metabolomics approach for the detection of the metabolites. Through PCA and OPLS-DA, the MNLC of scopoletin-treated group was clearly separated from the other two scopoletin treated groups (1/9 and 1/3 MNLC of scopoletin-treated groups). The 1/9 and 1/3 MNLC of scopoletin-treated groups were closer to the control group. Based on our standard for significantly differential metabolites, 60 metabolites were selected as potential biomarkers of scopoletin exposure. Pathway analyses showed that multiple metabolic pathways were affected by scopoletin exposure, including phosphonate and phosphinate metabolism, vitamin B6 metabolism, histidine metabolism, sphingolipid metabolism, lysine degradation, and folate biosynthesis. Metabolite levels in phosphonate and phosphinate metabolism, vitamin B6 metabolism, and histidine metabolism increased, while the metabolite in sphingolipid metabolism was reduced. The involved metabolic pathway network revealed the potential mechanism of the metabolic perturbation induced by scopoletin. This study provides a comprehensive metabolic profiling of scopoletin exposure, and those disturbed pathways could result in a biologically harmful effect on zebrafish embryos.

## Data Availability

Data available on request.

## Supplementary Materials

The following supporting information can be downloaded at: https://www.mdpi.com/article/10.3390/metabo12100934/s1, Figure S1: The reliability investigation of the analytical method using QC samples. Orange and red lines indicate the 2 SD and 3 SD limits of peak height intensities, respectively. (A) ESI−; (B) ESI+. Figure S2: PCA loading plot derived from UHPLC-MS/MS analysis for the solvent control, fish water control, and scopoletin exposure groups. Data were acquired by negative ionization (A) and positive ionization (B). In the PCA loading plots, metabolites with labeled name represent metabolites which caused the MNLC of scopoletin-treated group separation. Figure S3: Heatmap and hierarchical clustering of metabolites in untargeted metabolomics analysis in the current study in negative mode (A) and positive mode (B). Fish water control (group A), 1% DMSO solvent control (group B), 1/9 MNLC of scopoletin exposure (group C), 1/3 MNLC of scopoletin exposure (group D), MNLC of scopoletin exposure (group E). Figure S4: (A) Cluster analysis among experimental samples. (B) Pearson correlation between experimental samples. Fish water control (group A), 1% DMSO solvent control (group B), 1/9 MNLC of scopoletin exposure (group C), 1/3 MNLC of scopoletin exposure (group D), MNLC of scopoletin exposure (group E). Figure S5: OPLS-DA derived from UHPLC-MS/MS analysis for the solvent control, fish water control, and scopoletin exposure groups. Data were acquired by negative ionization (A) and positive ionization (B). In the OPLS-DA model, green points represent the fish water control group, blue points represent the solvent control group, red points represent the 1/9 MNLC of scopoletin-treated group, yellow points represent the 1/3 MNLC of scopoletin-treated group, and baby-blue points represent the MNLC of scopoletin-treated group. Figure S6: Variable importance in projection (VIP) analyses under negative mode. Each var ID represents a detected metabolite under negative mode (Supplementary Materials Excel 1). Figure S7: Variable importance in projection (VIP) analyses under positive mode. Each var ID represents a detected metabolite under positive mode (Supplementary Materials Excel 2). Table S1: Calculated 120-h MNLC (the maximum non-lethal concentration) and LC_10_ (lethal concentration 10%) values for scopoletin to zebrafish embryos. Data from our previous study. Table S2: Differential metabolites identified significantly different in zebrafish embryos induced by scopoletin.

## References

[1] Seo E.-J., Saeed M., Law B., Wu A., Kadioglu O., Greten H., Efferth T. (2016). Pharmacogenomics of Scopoletin in Tumor Cells. Molecules.

[2] Antika L.D., Tasfiyati A.N., Hikmat H., Septama A.W. (2022). Scopoletin: A review of its source, biosynthesis, methods of extraction, and pharmacological activities. Z. Naturforsch. C J. Biosci..

[3] Liu X.L., Zhang L., Fu X.L., Chen K., Qian B.C. (2001). Effect of scopoletin on PC3 cell proliferation and apoptosis. Acta Pharmacol. Sin..

[4] Shi Z., Li N., Chen C., Wang Y., Lei Z., Chen L., Sun J. (2020). Novel NO-releasing scopoletin derivatives induce cell death via mitochondrial apoptosis pathway and cell cycle arrest. Eur. J. Med. Chem..

[5] Pan R., Dai Y., Gao X.-H., Lu D., Xia Y.-F. (2010). Inhibition of vascular endothelial growth factor-induced angiogenesis by scopoletin through interrupting the autophosphorylation of VEGF receptor 2 and its downstream signaling pathways. Vascul. Pharmacol..

[6] Graña E., Costas-Gil A., Longueira S., Celeiro M., Teijeira M., Reigosa M.J., Sánchez-Moreiras A.M. (2017). Auxin-like effects of the natural coumarin scopoletin on Arabidopsis cell structure and morphology. J. Plant Physiol..

[7] Gao J., Wang F., Cui J., Zhang Q., Wang P., Liu D., Zhou Z. (2021). Assessment of toxicity and environmental behavior of chiral ethiprole and its metabolites using zebrafish model. J. Hazard. Mater..

[8] Wang X., Qin Y., Li X., Yan B., Martyniuk C.J. (2021). Comprehensive Interrogation of Metabolic and Bioenergetic Responses of Early-Staged Zebrafish (*Danio rerio*) to a Commercial Copper Hydroxide Nanopesticide. Environ. Sci. Technol..

[9] Weng Y., Huang Z., Wu A., Yu Q., Lu H., Lou Z., Lu L., Bao Z., Jin Y. (2021). Embryonic toxicity of epoxiconazole exposure to the early life stage of zebrafish. Sci. Total Environ..

[10] Lee H., Sung E.J., Seo S., Min E.K., Lee J.-Y., Shim I., Kim P., Kim T.-Y., Lee S., Kim K.-T. (2021). Integrated multi-omics analysis reveals the underlying molecular mechanism for developmental neurotoxicity of perfluorooctanesulfonic acid in zebrafish. Environ. Int..

[11] Merino C., Casado M., Piña B., Vinaixa M., Ramírez N. (2021). Toxicity of 4-(methylnitrosamino)-1-(3-pyridyl)-1-butanone (NNK) in early development: A wide-scope metabolomics assay in zebrafish embryos. J. Hazard. Mater..

[12] Patti G.J., Yanes O., Siuzdak G. (2012). Innovation: Metabolomics: The apogee of the omics trilogy. Nat. Rev. Mol. Cell Biol..

[13] Wang J., Lin L., Huang J., Zhang J., Duan J., Guo X., Wu S., Sun Z. (2022). Impact of PM2.5 Exposure on Plasma Metabolome in Healthy Adults during Air Pollution Waves: A Randomized, Crossover Trial. J. Hazard. Mater..

[14] Xiao Y., Wang Y.-K., Xiao X.-R., Zhao Q., Huang J.-F., Zhu W.-F., Li F. (2020). Metabolic profiling of coumarins by the combination of UPLC-MS-based metabolomics and multiple mass defect filter. Xenobiotica.

[15] Chen P., Yang J., Wang R., Xiao B., Liu Q., Sun B., Wang X., Zhu L. (2021). Graphene oxide enhanced the endocrine disrupting effects of bisphenol A in adult male zebrafish: Integrated deep learning and metabolomics studies. Sci. Total Environ..

[16] Zhao Q., Li X.-M., Liu H.-N., Gonzalez F.J., Li F. (2017). Metabolic map of osthole and its effect on lipids. Xenobiotica.

[17] Raldúa D., Casado M., Prats E., Faria M., Puig-Castellví F., Pérez Y., Alfonso I., Hsu C.-Y., Arick Ii M.A., Garcia-Reyero N. (2020). Targeting redox metabolism: The perfect storm induced by acrylamide poisoning in the brain. Sci. Rep..

[18] Lee H.-K., Kim K., Lee J., Lee J., Lee J., Kim S., Lee S.-E., Kim J.-H. (2020). Targeted toxicometabolomics of endosulfan sulfate in adult zebrafish (*Danio rerio*) using GC-MS/MS in multiple reaction monitoring mode. J. Hazard. Mater..

[19] Basu S., Duren W., Evans C.R., Burant C.F., Michailidis G., Karnovsky A. (2017). Sparse network modeling and metscape-based visualization methods for the analysis of large-scale metabolomics data. Bioinformatics.

[20] Martínez-Navarro F.J., Martínez-Morcillo F.J., López-Muñoz A., Pardo-Sánchez I., Martínez-Menchón T., Corbalán-Vélez R., Cayuela M.L., Pérez-Oliva A.B., García-Moreno D., Mulero V. (2020). The vitamin B6-dependent enzymes PYGL and G6PD fuel NADPH oxidases to promote skin inflammation. Dev. Comp. Immunol..

[21] Cabrini L., Bergami R., Fiorentini D., Marchetti M., Landi L., Tolomelli B. (1998). Vitamin B6 deficiency affects antioxidant defences in rat liver and heart. Biochem Mol Biol Int..

[22] Zhang C.-Y., Flor S., Ruiz P., Dhakal R., Hu X., Teesch L.M., Ludewig G., Lehmler H.-J. (2020). 3,3’-Dichlorobiphenyl Is Metabolized to a Complex Mixture of Oxidative Metabolites, Including Novel Methoxylated Metabolites, by HepG2 Cells. Environ. Sci. Technol..

[23] Eifel P.J., Brown D.M., Lee W.W., Brown J.M. (1983). Misonidazole neurotoxicity in mice decreased by administration with pyridoxine. Int. J. Radiat. Oncol. Biol. Phys..

[24] Slováčková J., Slavík J., Kulich P., Večeřa J., Kováč O., Paculová H., Straková N., Fedr R., Silva J.P., Carvalho F. (2021). Polychlorinated environmental toxicants affect sphingolipid metabolism during neurogenesis in vitro. Toxicology.

[25] Xu Y., Zhao Y., Guo X., Li Y., Zhang Y. (2018). Plasma metabolic profiling analysis of neurotoxicity induced by oxaliplatin using metabonomics and multivariate data analysis. Toxicol. Res..

[26] Alaamery M., Albesher N., Aljawini N., Alsuwailm M., Massadeh S., Wheeler M.A., Chao C.-C., Quintana F.J. (2020). Role of sphingolipid metabolism in neurodegeneration. J. Neurochem..

[27] Apostolopoulou M., Gordillo R., Koliaki C., Gancheva S., Jelenik T., De Filippo E., Herder C., Markgraf D., Jankowiak F., Esposito I. (2018). Specific Hepatic Sphingolipids Relate to Insulin Resistance, Oxidative Stress, and Inflammation in Nonalcoholic Steatohepatitis. Diabetes Care.

[28] DeVos L., Chanson A., Liu Z., Ciappio E.D., Parnell L.D., Mason J.B., Tucker K.L., Crott J.W. (2008). Associations between single nucleotide polymorphisms in folate uptake and metabolizing genes with blood folate, homocysteine, and DNA uracil concentrations. Am. J. Clin. Nutr..

[29] Kang S.-S., Wong P.W.K., Norusis M. (1987). Homocysteinemia due to folate deficiency. Metabolism.

[30] Yin J., Hong X., Ma L., Liu R., Bu Y. (2020). Non-targeted metabolomic profiling of atrazine in Caenorhabditis elegans using UHPLC-QE Orbitrap/MS. Ecotoxicol. Environ. Saf..

