# Supplementary material for: Scopoletin Induced Metabolomic Profile Disturbances in Zebrafish Embryos
Source: Metabolites. 2022 Oct 1;12(10):934. doi: 10.3390/metabo12100934 (PMC9609460; doi:10.3390/metabo12100934)
Supplement: Supplementary file 1 [file metabolites-12-00934-s001.zip › metabolites-1898801-Supplementary Material.pdf]

Supplementary Material

Scopoletin Induced Metabolomic Profile Disturbances in Zebrafish Embryos

Weixuan Yao [1,*], Jingpei Chen [1], Zhanyu Lin [1], Nani Wang [2], Anli Wang [3], Binjie Wang [1], Yuanzhao Wu [1], Zhongshi Xu [1] and Jiye Wang [1,*]

[1] Key Laboratory of Drug Prevention and Control Technology of Zhejiang Province, The Department of Criminal Science and Technology, Zhejiang Police College, Hangzhou 310053, China
[2] Department of Medicine, Zhejiang Academy of Traditional Chinese Medicine, Hangzhou 310012, China
[3] College of Biosystems Engineering and Food Science, Zhejiang University, Hangzhou 310058, China
* Correspondence: yaoweixuan@zjpc.edu.cn (W.Y.); wangjiye@zjjcxy.cn (J.W.)

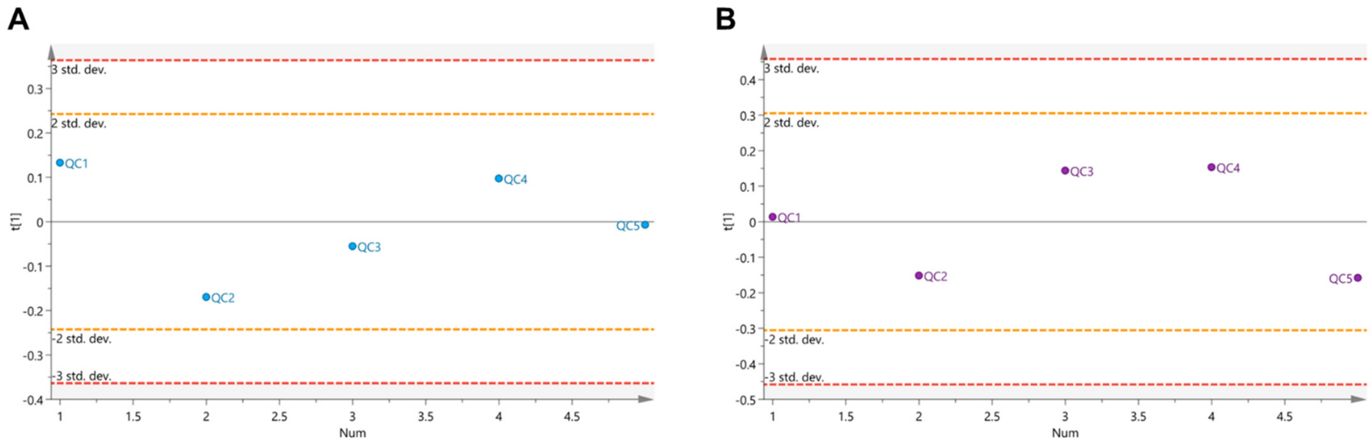

Figure S1. The reliability investigation of the analytical method using QC samples. Orange and red lines indicate the 2 SD and 3 SD limits of peak height intensities, respectively. (A) ESI−; (B) ESI+.

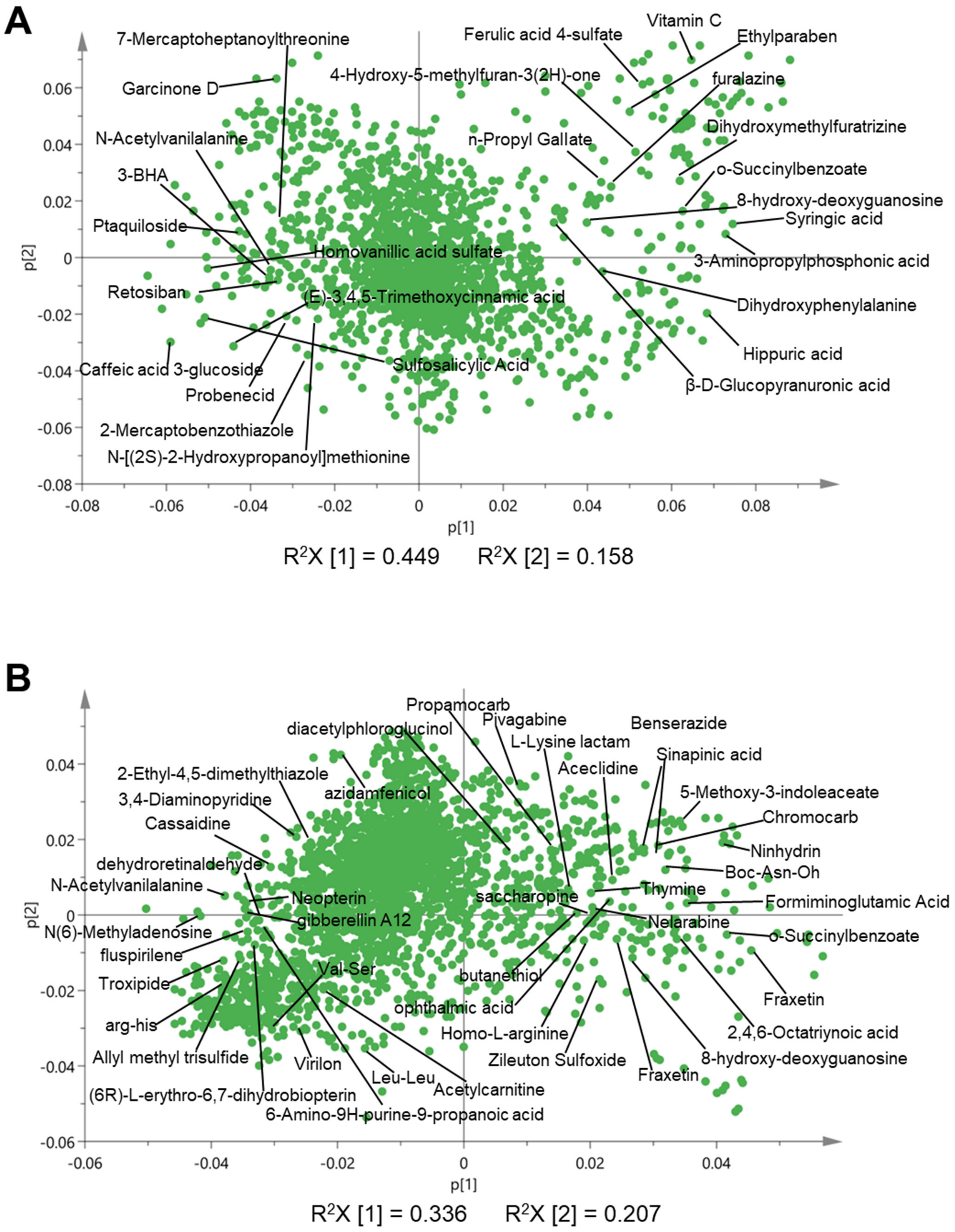

Figure S2. PCA loading plot derived from UHPLC-MS/MS analysis for the solvent control, fish water control, and scopoletin exposure groups. Data were acquired by negative ionization (A) and positive ionization (B). In the PCA loading plots, metabolites with labeled name represent metabolites which caused the MNLC of scopoletin-treated group separation.

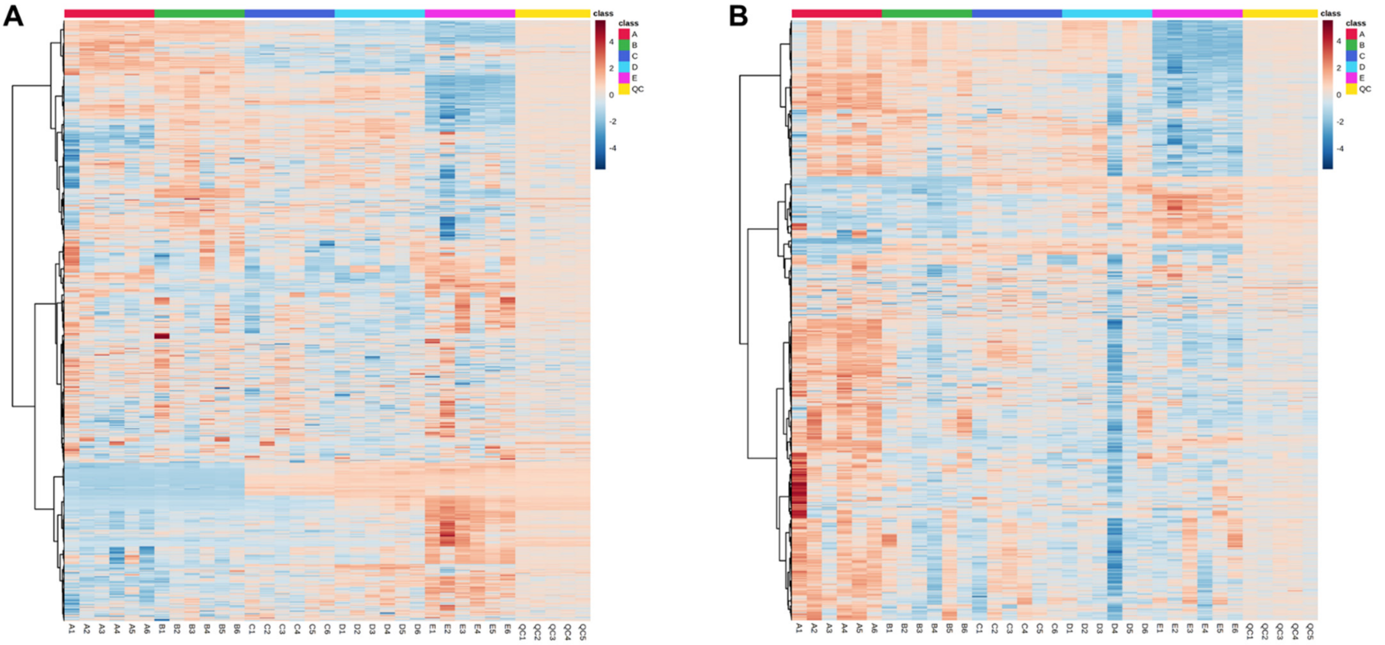

Figure S3. Heatmap and hierarchical clustering of metabolites in untargeted metabolomics analysis in the current study in negative mode (A) and positive mode (B). Fish water control (group A), 1% DMSO solvent control (group B), 1/9 MNLC of scopoletin exposure (group C), 1/3 MNLC of scopoletin exposure (group D), MNLC of scopoletin exposure (group E).

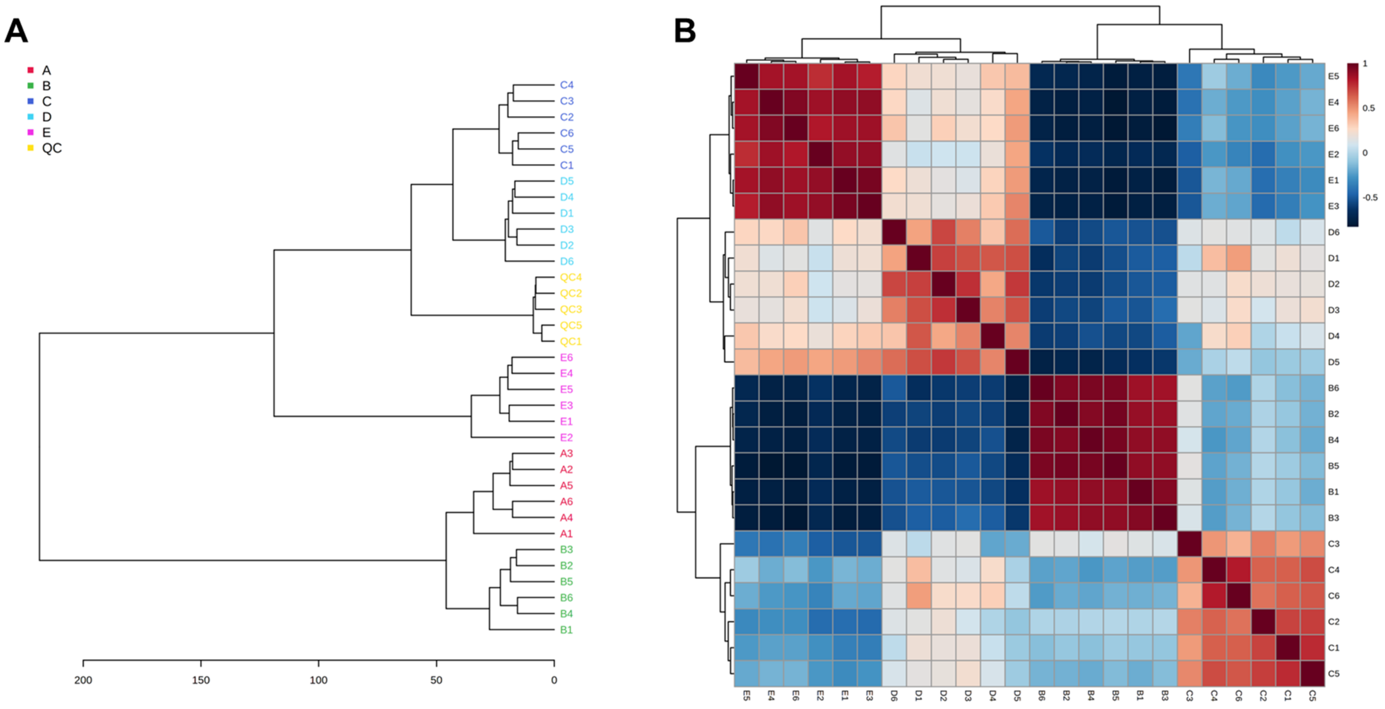

Figure S4. (A) Cluster analysis among experimental samples. (B) Pearson correlation between experimental samples. Fish water control (group A), 1% DMSO solvent control (group B), 1/9 MNLC of scopoletin exposure (group C), 1/3 MNLC of scopoletin exposure (group D), MNLC of scopoletin exposure (group E).

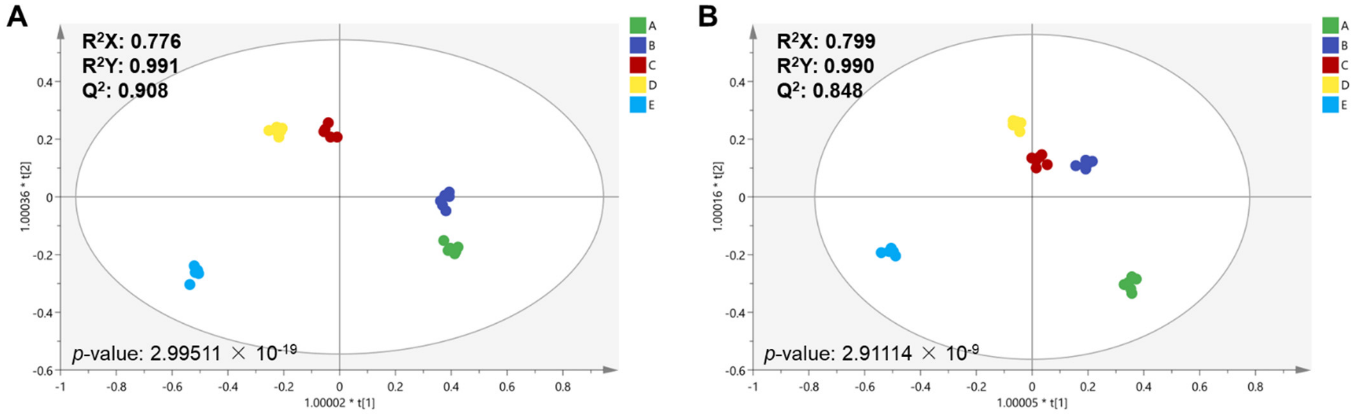

Figure S5. OPLS-DA derived from UHPLC-MS/MS analysis for the solvent control, fish water control, and scopoletin exposure groups. Data were acquired by negative ionization (A) and positive ionization (B). In the OPLS-DA model, green points represent the fish water control group, blue points represent the solvent control group, red points represent the 1/9 MNLC of scopoletin-treated group, yellow points represent the 1/3 MNLC of scopoletin-treated group, and baby-blue points represent the MNLC of scopoletin-treated group.

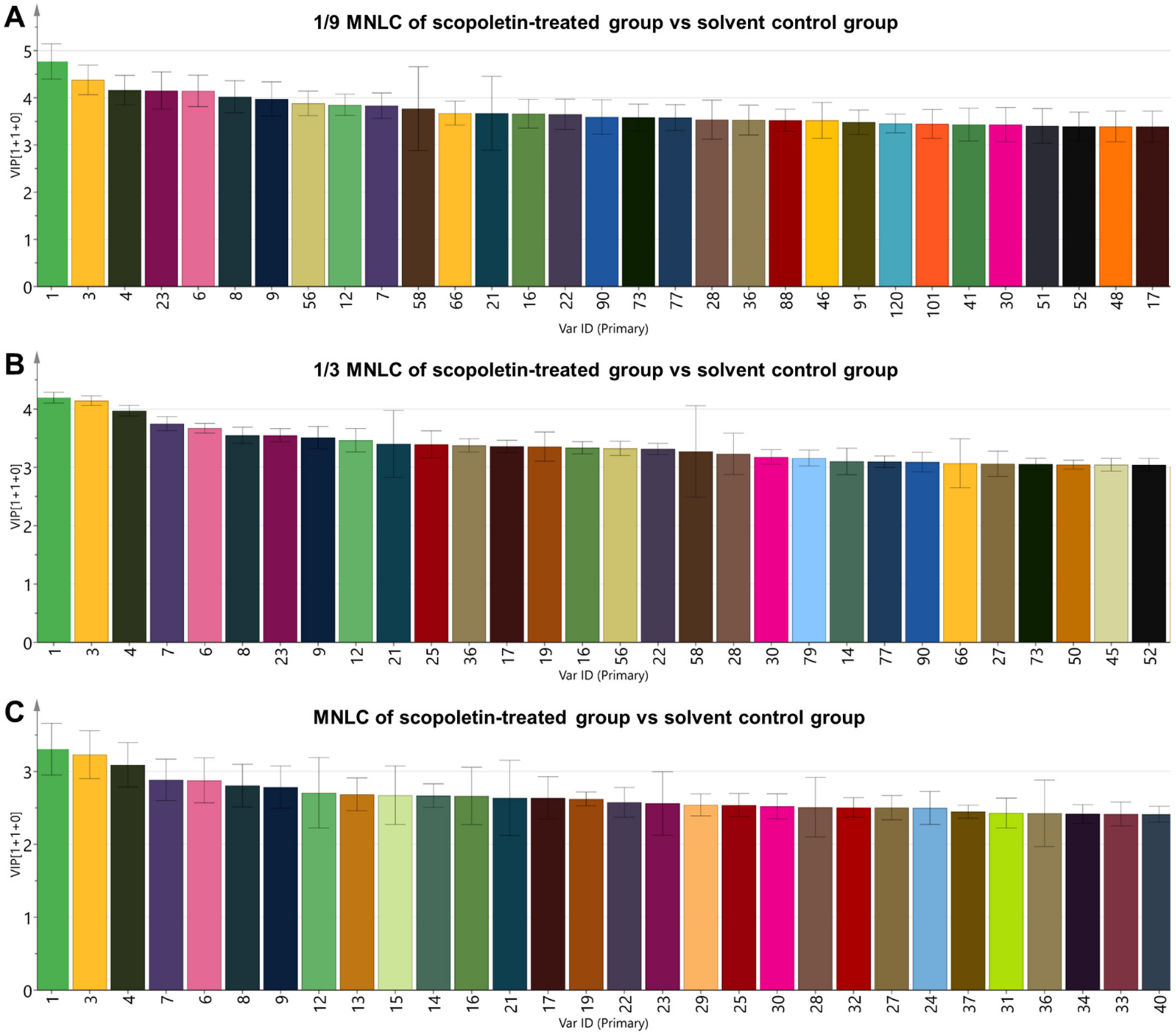

Figure S6. Variable importance in projection (VIP) analyses under negative mode. Each var ID represents a detected metabolite under negative mode (Supplementary Materials Excel 1).

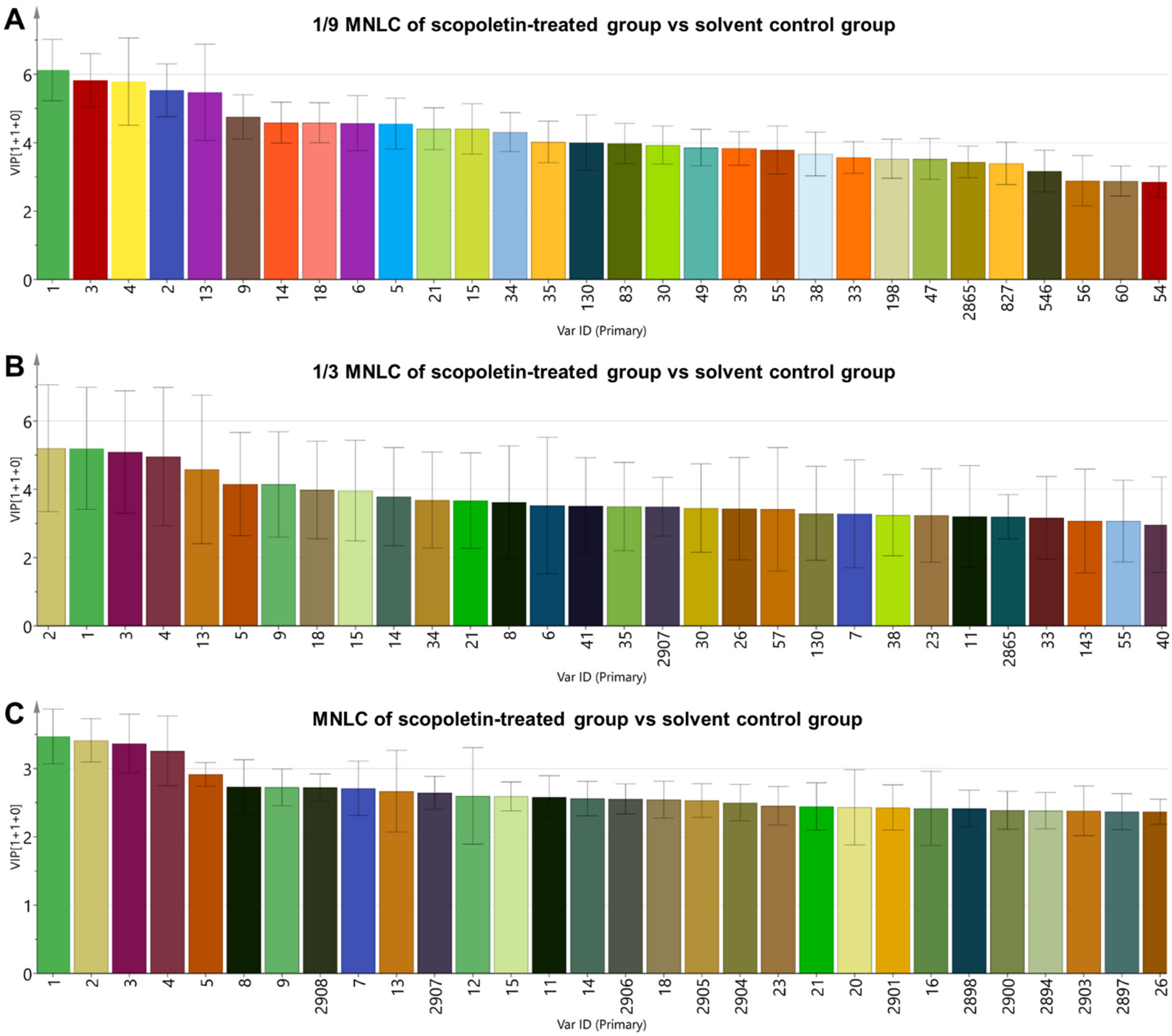

Figure S7. Variable importance in projection (VIP) analyses under positive mode. Each var ID represents a detected metabolite under positive mode (Supplementary Materials Excel 2).

Table S1. Calculated 120-h MNLC (the maximum non-lethal concentration) and LC_{10} (lethal concentration 10%) values for scopoletin to zebrafish embryos. Data from our previous study.

Chemical exposure	Calculated 120-h MNLC	Calculated 120-h LC_{10}
scopoletin	18.5 μg/mL	25.8 μg/mL

Table S2. Differential metabolites identified significantly different in zebrafish embryos induced by scopoletin.

No.	Putative Identification	Log_2 Fold Change: (C) / (B)	*P*-value : (C) / (B)	Log_2 Fold Change: (D) / (B)	*P*-value : (D) / (B)	Log_2 Fold Change: (E) / (B)	*P*-value : (E) / (B)
1	(6R)-L-erythro-6,7-dihydrobiopterin	-1.04	7.29233E-05	-1.45	1.9558E-05	-2.12	6.28192E-07
2	(all-Z)-20-hydroxy-5,8,11,14-Eicosatetraenoic acid	0.84	0.00021318	1.67	6.4887E-05	1.39	0.000568172
3	2,4,6-Octatriynoic acid	4.29	6.02487E-08	4.71	1.07999E-06	4.22	1.57429E-08
4	2-Ethyl-4,5-dimethylthiazole	-0.44	0.262760126	-0.68	0.089249574	-1.06	0.021196787
5	2-Mercaptobenzothiazole	-1.80	2.0989E-11	-1.64	1.83273E-08	-2.21	5.48273E-07
6	2-Aminoethylphosphonate	5.03	0.001948102	8.15	6.67192E-05	10.70	0.002242889
7	3-BHA	-1.49	5.60144E-05	-2.29	2.98864E-06	-3.29	5.00857E-07
8	4-Hydroxy-5-methylfuran-3(2H)-one	4.92	1.21609E-12	5.27	2.48849E-10	4.92	4.01504E-09
9	5-Methoxy-3-indoleaceate	1.19	0.131504053	0.75	0.065537092	5.05	0.042050459
10	6-Amino-9H-purine-9-propanoic acid	-0.72	0.003245215	-1.42	0.000502246	-1.77	3.70279E-05
11	7-Mercaptoheptanoylthreonine	-1.57	0.000108869	-1.55	0.000138545	-1.94	6.48335E-05
12	8-hydroxy-deoxyguanosine	2.10	6.9032E-05	2.88	1.83554E-05	2.87	7.20275E-09
13	Aceclidine	0.82	0.004014541	1.03	0.01762538	2.73	2.74474E-06
14	Acetylcarnitine	-0.62	0.048283784	-0.79	0.02347808	-3.15	9.15784E-06
15	Allyl methyl trisulfide	-1.40	2.80794E-06	-1.91	1.54798E-07	-3.04	1.14361E-08
16	arg-his	-0.79	0.010138861	-0.64	0.036982522	-3.21	4.06953E-05
17	butanethiol	1.22	1.38043E-05	1.51	0.000483328	1.60	7.42141E-06
18	Caffeic acid 3-glucoside	-1.94	0.000219329	-3.09	9.57257E-07	-3.75	3.90426E-07
19	Cassaidine	-1.06	0.032912812	-0.72	0.128794609	-1.30	0.014510268
20	dehydroretinaldehyde	-0.80	0.001204627	-1.05	0.000175231	-1.92	1.82451E-06
21	diacetylphloroglucinol	4.10	5.24763E-06	2.00	0.006343706	2.08	0.017443437
22	dihydroxyphenylalanine	1.35	1.78447E-05	2.51	7.96312E-09	3.99	2.03284E-07
23	Ethylparaben	4.09	0.000954175	3.81	5.50473E-05	3.71	2.96107E-08
24	Ferulic acid 4-sulfate	8.27	2.2184E-07	7.80	7.69194E-07	6.42	5.43885E-06
25	fluspirilene	-0.61	0.121121888	1.14	0.019796957	-3.05	0.000447627

26	Fraxetin	5.25	5.18731E-05	8.08	0.005345636	7.52	0.007150002
27	furalazine	1.96	1.12027E-08	2.87	2.83456E-10	3.08	9.43764E-11
28	Garcinone D	-1.18	9.09903E-06	-2.30	2.36327E-07	-6.58	1.99031E-08
29	gibberellin A12	-0.53	0.035543878	-1.32	0.000175142	-2.34	1.27007E-05
30	Hippuric acid	1.48	0.001099191	2.15	0.000488642	2.40	0.016116525
31	Homo-L-arginine	0.67	0.000671632	1.01	0.000270311	2.34	0.000192519
32	Homovanillic acid sulfate	-3.63	3.92194E-10	-3.84	3.28506E-10	-5.81	1.85849E-10
33	Leu-Leu	-0.94	0.014097761	-0.68	0.063437702	-4.22	9.75403E-05
34	L-Lysine lactam	1.48	0.00223666	1.79	0.000331936	1.95	0.001857999
35	N (6)-Methyladenosine	-1.29	2.848E-06	-2.65	7.27781E-08	-4.16	1.35191E-08
36	N-Acetylvanilalanine	-0.87	0.000582236	-1.94	1.21124E-05	-2.92	2.4425E-06
37	Nelarabine	0.64	0.090417341	1.06	0.01400039	1.85	0.021738996
38	Neopterin	-0.53	0.109254231	-1.36	0.004852523	-2.49	0.000449367
39	N-Formimino-L-glutamate	1.25	0.001781641	1.99	0.010611658	3.65	0.002855792
40	Ninhydrin	0.52	0.006128159	2.22	0.04203568	6.36	0.077793053
41	n-Propyl GalIate	4.49	0.000731944	3.42	1.68782E-07	4.64	5.0318E-05
42	ophthalmic acid	0.72	4.56019E-05	1.48	0.000234974	2.11	8.30937E-08
43	o-Succinylbenzoate	0.86	0.003874337	4.71	0.001849356	5.23	9.77297E-05
44	Phytoceramide	-0.80	0.059241345	-1.18	0.017630395	-1.47	0.011169334
45	Pivagabine	0.63	0.022788804	0.80	0.05479367	2.56	3.56304E-07
46	Probenecid	-1.18	5.37255E-06	-1.69	8.63695E-07	-2.17	2.67696E-07
47	Propamocarb	0.43	0.000345825	0.61	0.005385523	1.84	0.007580298
48	Ptaquiloside	-1.58	2.49375E-05	-3.04	8.13515E-07	-4.90	3.12617E-07
49	Pyridoxine	1.96	2.28312E-07	2.63	0.000193004	2.58	2.58829E-07
50	saccharopine	0.99	0.004592472	0.74	0.023149175	1.44	2.96373E-05
51	Sinapinic acid	5.17	3.57294E-07	5.69	6.34611E-05	3.19	0.000520244
52	Sulfosalicylic Acid	-4.12	1.61125E-08	-4.11	1.69491E-08	-4.45	2.72541E-08
53	Syringic acid	5.35	0.001144395	8.33	6.19288E-05	10.75	0.001745967

54	Thymine	1.04	0.021680173	0.80	0.013073583	2.03	0.023997159
55	Troxipide	-0.94	0.009056559	-0.80	0.026921682	-3.78	5.26555E-05
56	Val-Ser	-0.44	0.213600646	0.67	0.100610393	-3.59	0.000519145
57	Virilon	2.45	5.1342E-06	1.04	0.016184979	-2.84	0.001283174
58	Vitamin C	9.73	2.66129E-12	10.06	4.36153E-11	9.72	9.11349E-09
59	Zileuton Sulfoxide	1.77	0.014198129	3.21	0.002718232	2.01	0.085754643
60	β-D-Glucopyranuronic acid	1.41	3.49632E-06	1.53	3.57553E-08	1.53	0.000275583

[1] Group B, 1% DMSO solvent control; Group C, 1/9 MNLC of scopoletin-treated group; Group D, 1/3 MNLC of scopoletin-treated group; Group E, MNLC of scopoletin-treated group.
